# Data supporting Maastrichtian paleoclimate variables applying a multi proxy approach to a paleosol profile, Arctic Alaska

**DOI:** 10.1016/j.dib.2020.105191

**Published:** 2020-01-25

**Authors:** Susana Salazar-Jaramillo, Paul J. McCarthy, Andrés Ochoa, Sarah J. Fowell, Fred J. Longstaffe

**Affiliations:** a Universidad Nacional de Colombia, Escuela de Geociencias, Calle 59 A No. 63 - 20 Bloque 14-215, Medellín, Colombia; b University of Alaska Fairbanks, Department of Geosciences, and Geophysical Institute, Fairbanks, AK, 99775-5780, USA; c Universidad Nacional de Colombia, Departamento de Geociencias y Medio Ambiente, Cl. 80 Nº 65 – 223, Medellín, Colombia; d University of Alaska Fairbanks, Department of Geosciences, Fairbanks, AK, 99775-5780, USA; e The University of Western Ontario, Department of Earth Sciences, London, Ontario, N6A 5B7, Canada

**Keywords:** Maastrichtian paleo-Arctic, Paleoclimate, Meteoric water composition, Silicate weathering

## Abstract

We provide the dataset of climate variables related to the research article “Paleoclimate reconstruction of the Prince Creek Formation, Arctic Alaska, during Maastrichtian global warming” [1]. The dataset includes mean annual precipitation (MAP) values determined using two independent proxies, estimates of the oxygen isotope composition of meteoric water (δ^18^O_w_) obtained from smectites and a comparison with previously published siderite data. We also provide the data used to calculate the total flux of CO_2_ required for the weathering of silicates. This dataset is an example of a multi proxy approach that could add further insight for researchers in the selection of suitable proxies for paleoclimatic interpretations.

**Table:** Specifications Table

Subject	Earth and Planetary Sciences: Earth-Surface Processes
Specific subject area	Paleoclimatology of a Maastrichtian paleosol profile in the paleo-Arctic using geochemical proxies.
Type of data	Tables Figures Raw data
How data were acquired	*XRF:* PANalytical Axios wavelength-dispersive X-ray fluorescence spectrometer (WD-XRF); *mass spectrometry* for *δ*^13^C: EA-IRMS (Elemental Analysis - Isotope Ratio Mass Spectrometry), Elemental Analyzer (ECS 4010); *mass spectrometry* for *δ*^18^O: Micromass Optima dual-inlet, IRMS; *software*: IDL. Details on analytical techniques in Ref. [1] and the section Experimental Design, Materials, and Methods.
Data format	Raw and analized data are provided in Table 1, Table 2, Table 3, Table 3, Table 4, Table 5 and in excel files as supplementary material.
Parameters for data collection	Field collection of bulk paleosol samples from a paleosol profile near the Kikak-Tegoseak dinosaur bonebed from bluffs along the Colville River, Alaska.
Description of data collection	Data collected included pedologic and sedimentologic macrostructures and microstructures, color, major oxide geochemistry, and total organic carbon (TOC) of paleosol samples. Details of the profile description are given in Ref. [2]. For the detailed geochemical analysis, one paleosol profile and two bentonite layers (NKT) were chosen. Samples were carefully taken every 20 cm after soil profile description according to Ref. [2]. Samples were air-dried and analysed according to the analytical techniques we describe in the section Experimental Design, Materials, and Methods.
Data source location	*Colville River, Alaska, U.S.,* paleosol profile: *N 69° 45.068′; W 151° 30.873′*; bentonites *N 69° 38.651’; W 151° 50.565′* and *N 69° 41.012′; W 151° 30.481′*
Data accessibility	All data are available within this article.
Related research article	Associated Paper: Salazar-Jaramillo S., McCarthy P.J., Ochoa A., Fowell, S.J., Longstaffe, F.J. Paleoclimate reconstruction of the Prince Creek Formation, Arctic Alaska, during Maastrichtian global warming. Palaeogeogr. Palaeoclimatol. Palaeoecol. 2019; 532 (July):109265. https://doi.org/10.1016/j.palaeo.2019.109265.

**Table:** 

**Value of the Data** • The climatic variables provide information regarding the Cretaceous paleo-Arctic that can be compared with previously published independent qualitative and quantitative data. • The data allow assessment of Cretaceous General Circulation Model (GCM) simulations through data-model comparisons. • The carbon isotopic composition (*δ*^13^C), obtained in paleosols from pollen and bulk organic matter, is valuable as a reference to refine proxies used in the identification of carbon cycle perturbations. • The oxygen isotopic composition of meteoric water (*δ*^18^O_w_) derived from smectite provides information as a paleohydrologic indicator, extending the sampling of high latitude continental deposits to pedogenic clays. • The total flux of CO_2_ required for silicate weathering is useful to understand the CO_2_ sinks in the geological carbon cycle in Maastrichtian arctic paleosols.

## 1 Data description

**Fig. 1:**
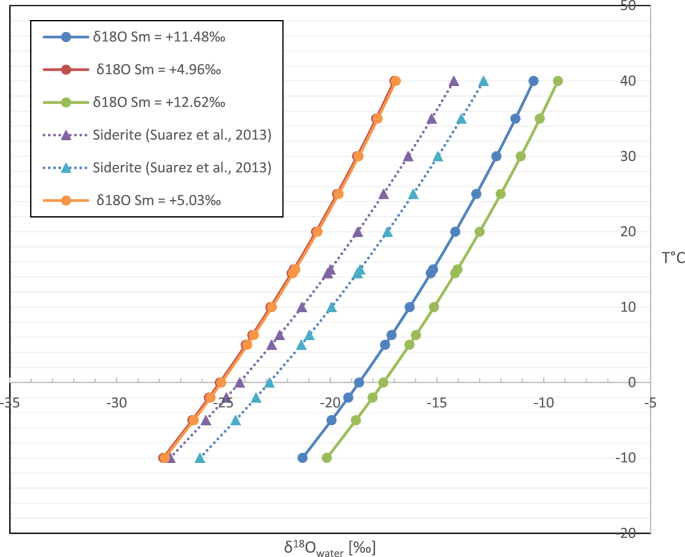
Solid lines show the isotopic composition of meteoric water derived from smectites based on the isotopic fractionation between smectite and meteoric water [4] using the maximum and minimum *δ*^18^O of bulk clay and bentonites. The dashed lines correspond to the meteoric water isotopic composition calculated using pedogenic siderite [5].

**Fig. 2:**
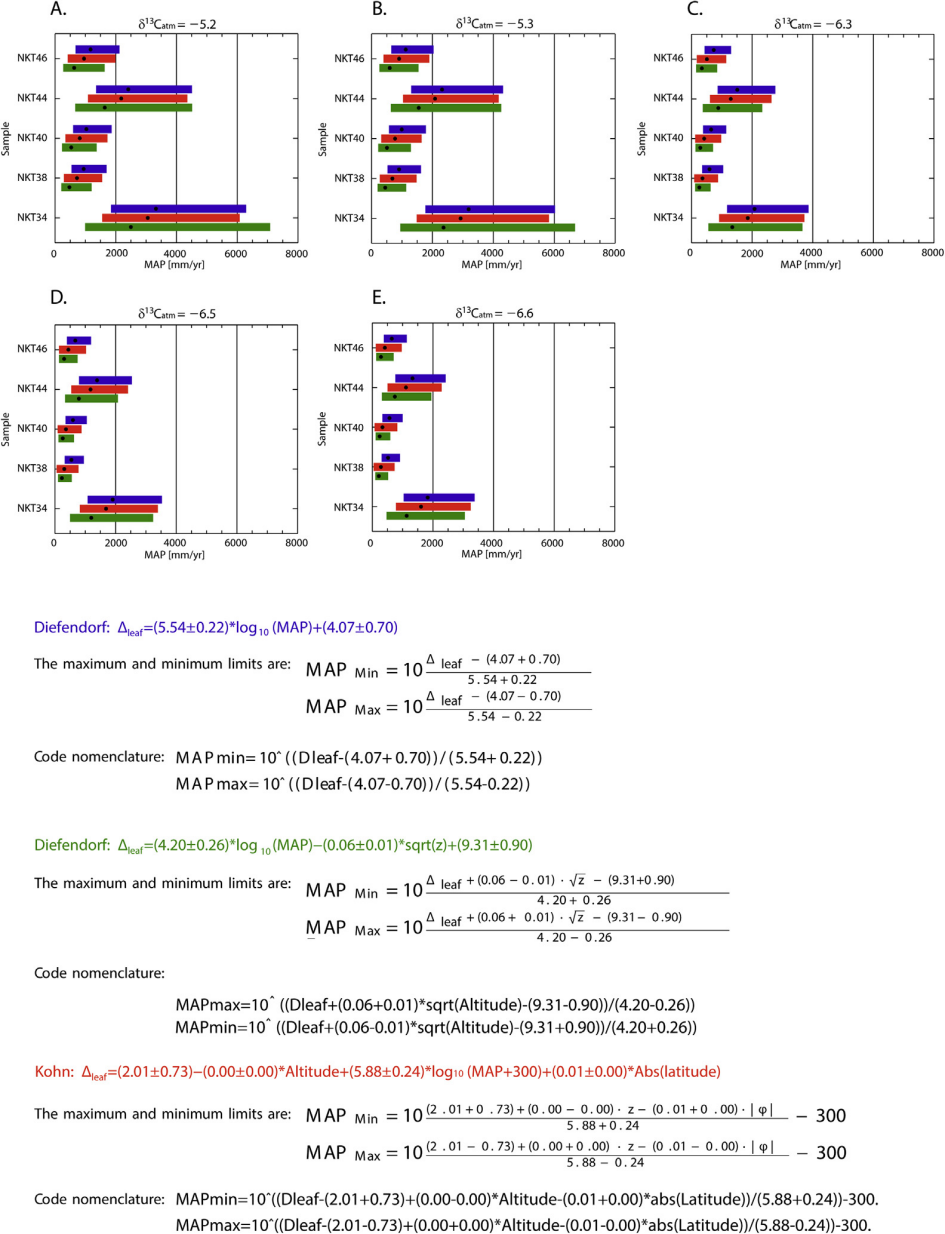
MAP variability in the Prince Creek Formation using *δ*^13^C_atm_ from several authors (Table 3 in Ref. [1]) in the [6,7] equations. A-E) Mean annual precipitation (MAP) using minimum and maximum values as indicated in the equations, where the black dot is the mean value. At the top of each plot are the corresponding *δ*^13^C_atm_ values used. The colors are related to the equation being used.

**Fig. 3:**
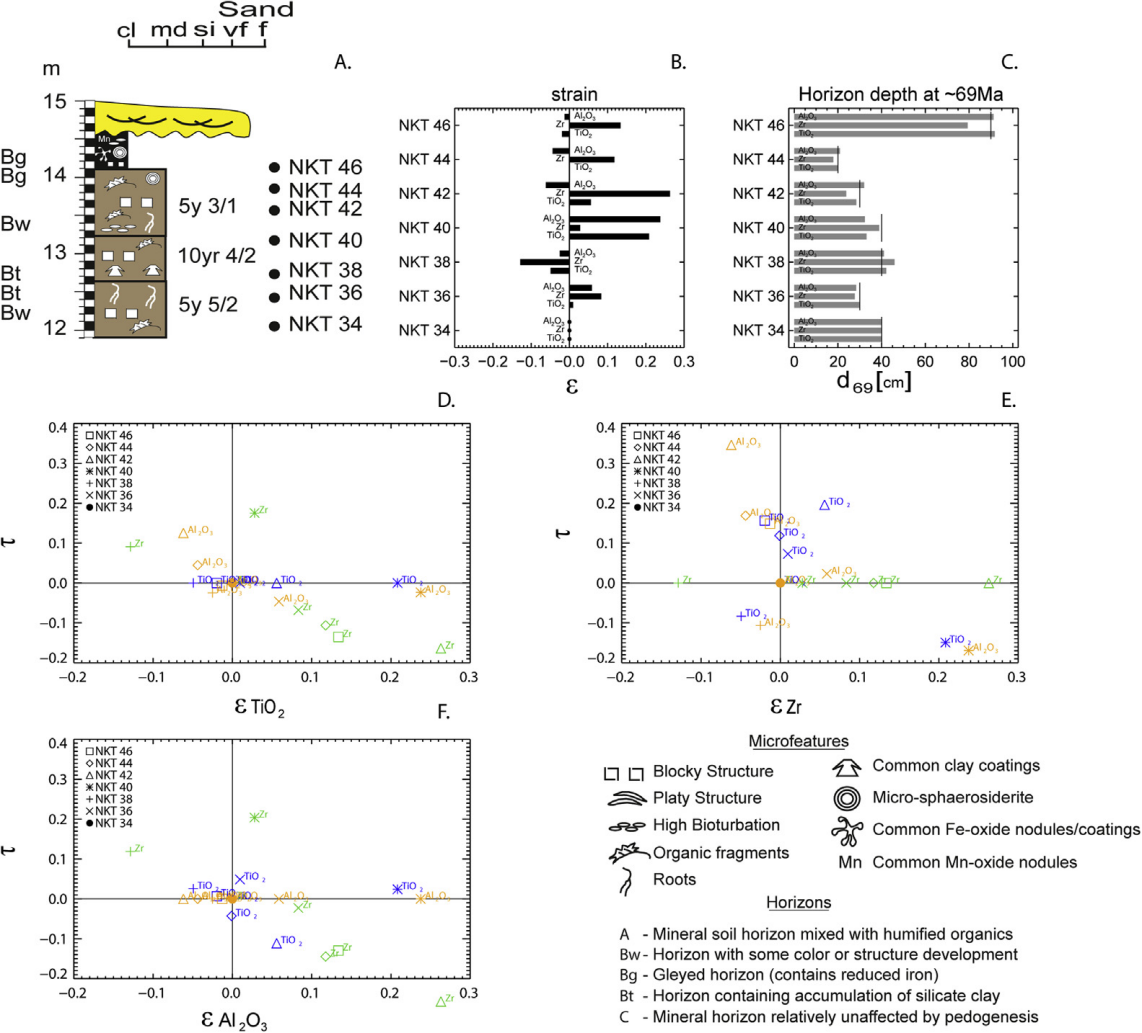
A) NKT paleosol horizons and depth. B) Strain (Ɛ). Volume change during weathering [8] calculated for the NKT paleosol. C) Thickness after applying a paleosol compaction equation at ∼69 Ma [15]. (D–E) Mass balance cross-plots of strain (ε) vs. elemental mass transport (τ) for the NKT paleosol using TiO_2_ (blue), Zr (green), and Al_2_O_3_ (yellow). The calculations assume immobility of D) TiO_2_, E) Zr, and F) Al_2_O_3_.

**Fig. 4:**
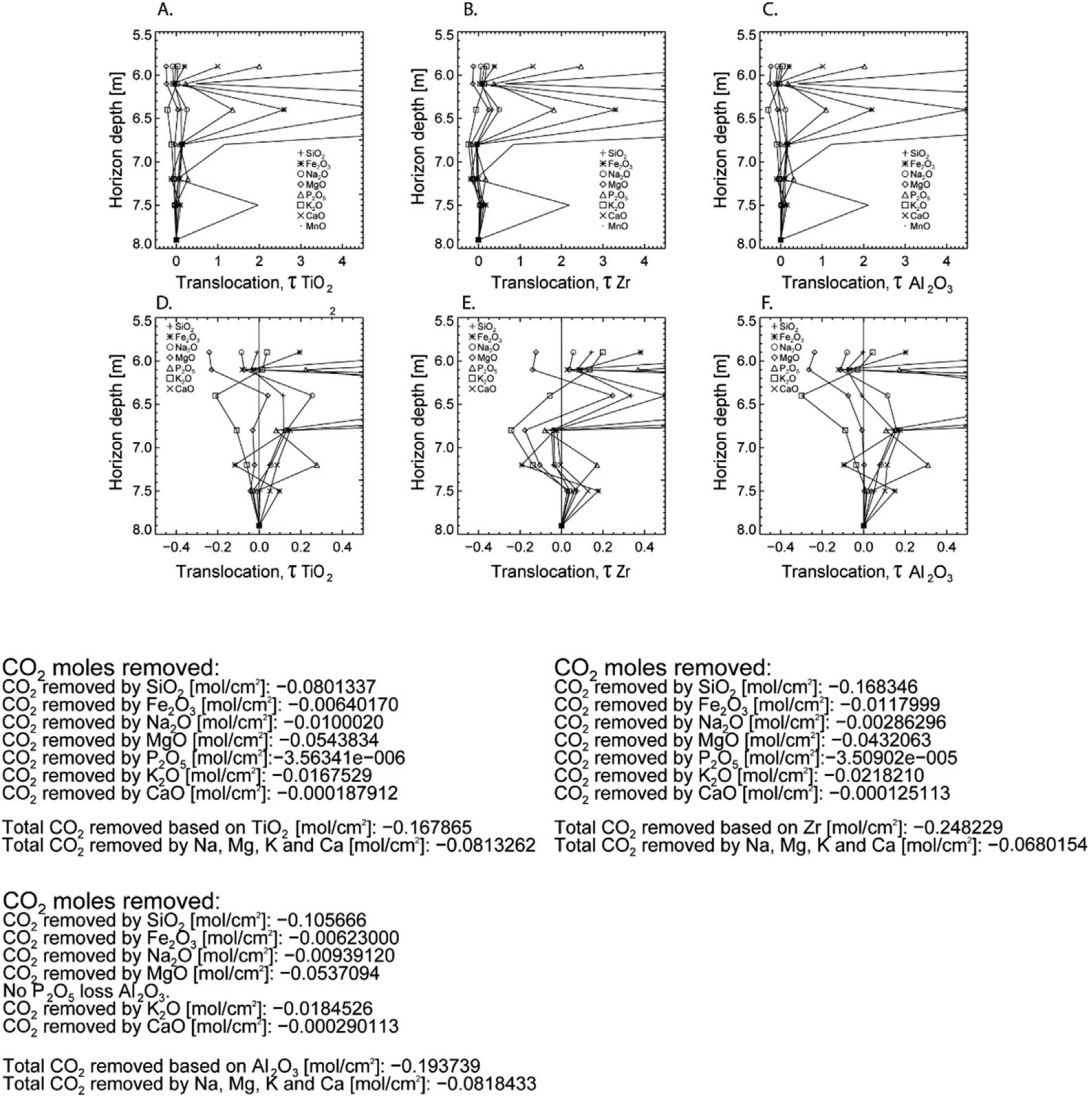
Elemental translocation (transport) assuming immobile A) TiO_2_, B) Zr, and C) Al_2_O_3_. Images (D–F) are the same translocations without the element Mn. At the bottom of the figure are mass-balance calculations that relate elemental weathering to atmospheric CO_2_ levels (given in [mol/cm^2^]) [9,10].

In this article, we report raw data of carbon and nitrogen isotopes (from pollen and bulk organic matter) in Table 1. Data of major elements from a Maastrichtian paleosol profile (n = 7) are reported in Table 2. These data are reported as raw, and as molar proportions, for the A-C*N-K diagram plotted in Ref. [1], using Al_2_O_3_, CaO* + Na_2_O, K_2_O, according to Ref. [3]. Both, raw and analysed oxygen isotope data from the total clay-size fraction for the NKT paleosol (n = 7) and from the KKT and PFDV bentonites (n = 2) are reported in Table 3, Table 4, respectively. Table 4 and Fig. 1 also report the calculated *δ*^18^O_w_ (‰. VSMOW) of meteoric water using the equation of Sheppard and Gilg [4], and compare the data with *δ*^18^O_w_ from siderite [5]. Fig. 2 is a plot of mean annual precipitation using three equations (and several *δ*^13^C atmospheric scenarios, [1]) that estimate MAP from *δ*^13^C in pollen [6,7]. Table 5 and Fig. 3 show analysed data for mass balance calculations [8], and Fig. 4 indicates mass-balance calculations that relate elemental weathering to atmospheric CO_2_ levels (given in [mol/cm^2^]) [9,10].

**Table 1:** Nitrogen (N), organic carbon (C), *δ*^15^N, *δ*^13^C and C/N ratio values of bulk samples (NKT) and palynologic separates (NKT-P).

Sample Name	Conc N	Conc C	*δ*^15^N	*δ*^13^C	C/N
			At–air	VPDB	
	(%)	(%)	(‰)	(‰)	
NKT34	0.12	1.21	+3.03	−27.45	9.80
NKT36					
NKT38	0.08	0.26	+2.58	−25.23	3.28
NKT40	0.07	0.31	+2.71	−25.19	4.17
NKT42					
NKT44	0.14	0.71	+2.72	−25.95	4.98
NKT46	0.10	0.54	+2.30	−25.03	5.53
Average	0.10	0.61	+2.67	−25.77	5.55
Stand.dev.	0.03	0.38	0.26	1.00	2.52
NKT34–P	1.49	47.28	+3.02	−28.12	31.63
NKT36–P	1.38	55.82	+2.43	−25.61	40.41
NKT38–P	1.41	59.03	+2.48	−25.24	41.95
NKT40–P	0.95	33.99	+2.76	−25.44	35.88
NKT42–P	0.64	19.65	+4.83	−27.48	30.48
NKT44–P	1.20	47.83	+3.51	−27.39	39.80
NKT46–P	1.43	50.38	+2.27	−25.73	35.18
Average	1.22	44.85	+3.04	−26.43	36.48
Stand.dev.	0.31	13.66	0.89	1.19	4.43

**Table 2:** Geochemical XRF raw data (in weight percent) and calculated CIA-K values [11] for the NKT paleosol.

sample	NKT 34	NKT 36	NKT 38	NKT 40	NKT 42	NKT 44	NKT 46
depth, m	12.1	12.5	12.8	13.2	13.6	13.9	14.1
SiO_2_	69.240	70.170	71.090	71.600	65.610	67.960	68.520
TiO_2_	0.930	0.940	0.910	0.860	0.790	0.950	0.930
Al_2_O_3_	17.710	17.070	16.900	15.990	16.930	18.900	17.590
Fe_2_O_3_	4.680	5.190	4.040	4.920	14.300	4.660	5.590
MnO	0.020	0.060	0.020	0.040	0.270	0.030	0.130
MgO	1.820	1.760	1.740	1.630	1.700	1.880	2.000
CaO	0.160	0.170	0.170	0.170	0.840	0.150	0.320
Na_2_O	1.510	1.480	1.560	1.580	1.610	1.430	1.380
K_2_O	3.000	2.930	2.760	2.470	2.010	3.110	3.110
P_2_O_5_	0.040	0.040	0.050	0.040	0.080	0.050	0.120
LOI	0.811	0.334	0.241	0.214	0.253	0.913	0.550
Sum	99.921	100.144	99.481	99.514	104.393	100.033	100.240
Molar proportions [3]							
CaO* (mol)	0.002	0.002	0.002	0.002	0.013	0.002	0.003
Al_2_O_3_ (mol)	0.174	0.167	0.166	0.157	0.166	0.185	0.173
Na_2_O (mol)	0.024	0.024	0.025	0.025	0.026	0.023	0.022
K_2_O (mol)	0.032	0.031	0.029	0.026	0.021	0.033	0.033
A-C*N**-**K							
A	0.749	0.746	0.746	0.745	0.733	0.763	0.748
C*N	0.113	0.116	0.122	0.131	0.173	0.101	0.109
K	0.137	0.139	0.132	0.124	0.094	0.136	0.143
Sum	1.000	1.000	1.000	1.000	1.000	1.000	1.000
CIA-K	86.860	86.570	85.980	85.042	80.949	88.295	87.275

**Table 3:** Calculated *δ*^18^O (‰ VSMOW) of meteoric water from the total clay-size fraction for the NKT paleosol and the KKT and PFDV bentonites. The last column is the calculated *δ*^18^O of meteoric water using temperatures (−2 °C minimum, 6.3 °C average, and 14.5 °C maximum) determined from CLAMP analysis of paleobotanical specimens [14].

Sample	T (C)	T (K)	2.55 × (10^6^/T^2^) – 4.05 [4]	δ^18^O Smectite (‰)	δ^18^O of Meteoric water
NKT-34	−2.00	271.15	30.63	12.62	−18.01
	6.30	279.45	28.60	12.62	−15.98
	14.50	287.65	26.77	12.62	−14.15
NKT-36	−2.00	271.15	30.63	11.86	−18.77
	6.30	279.45	28.60	11.86	−16.74
	14.50	287.65	26.77	11.86	−14.91
NKT-38	−2.00	271.15	30.63	11.65	−18.98
	6.30	279.45	28.60	11.65	−16.95
14.50	287.65	26.77	11.65	−15.12	
NKT-40	−2.00	271.15	30.63	11.48	−19.15
	6.30	279.45	28.60	11.48	−17.12
	14.50	287.65	26.77	11.48	−15.29
NKT-42	−2.00	271.15	30.63	12.36	−18.27
	6.30	279.45	28.60	12.36	−16.24
	14.50	287.65	26.77	12.36	−14.41
NKT-44	−2.00	271.15	30.63	12.28	−18.35
	6.30	279.45	28.60	12.28	−16.32
	14.50	287.65	26.77	12.28	−14.49
NKT-46	−2.00	271.15	30.63	12.17	−18.46
	6.30	279.45	28.60	12.17	−16.43
	14.50	287.65	26.77	12.17	−14.60
D6KKT-20.5	−2.00	271.15	30.63	4.96	−25.67
	6.30	279.45	28.60	4.96	−23.64
	14.50	287.65	26.77	4.96	−21.81
PFDV-17-5.7	−2.00	271.15	30.63	5.03	−25.60
	6.30	279.45	28.60	5.03	−23.57
	14.50	287.65	26.77	5.03	−21.74

**Table 4:** Calculated *δ*^18^O (‰ VSMOW) of meteoric water derived from the Prince Creek Formation at several temperatures. Data from smectites (bentonites and total clay) are estimated based on the fractionation equation of [4]. In order to compare with published values, the siderite data are taken from Ref. [5], which uses the fractionation equation of [12].

T (C)	T (K)	1000 Ln α_x-w_		δ^18^O of Meteoric water using the following: δ^18^O Sid and δ^18^O Sm values (SMOW)					
		3.13 × (10^6^/T^2^) – 3.5	2.55 × (10^6^/T^2^) – 4.05	δ^18^O Sid = +14.21‰	δ^18^O Sid = +15.60‰	δ^18^O Sm = +4.96‰	δ^18^O Sm = +5.03‰	δ^18^O Sm = +11.48‰	δ^18^O Sm = +12.62‰
		[12]	[4]	[5]		This work			
−14.50	258.65	43.29	34.07	−29.08	−27.69	−29.11	−29.04	−22.59	−21.45
−10.00	263.15	41.70	32.77	−27.49	−26.10	−27.81	−27.74	−21.29	−20.15
−5.00	268.15	40.03	31.41	−25.82	−24.43	−26.45	−26.38	−19.93	−18.79
−2.00	271.15	39.07	30.63	−24.86	−23.47	−25.67	−25.60	−19.15	−18.01
0.00	273.15	38.45	30.13	−24.24	−22.85	−25.17	−25.10	−18.65	−17.51
5.00	278.15	36.96	28.91	−22.75	−21.36	−23.95	−23.88	−17.43	−16.29
6.30	279.45	36.58	28.60	−22.37	−20.98	−23.64	−23.57	−17.12	−15.98
10.00	283.15	35.54	27.76	−21.33	−19.94	−22.80	−22.73	−16.28	−15.14
14.50	287.65	34.33	26.77	−20.12	−18.73	−21.81	−21.74	−15.29	−14.15
15.00	288.15	34.20	26.66	−19.99	−18.60	−21.70	−21.63	−15.18	−14.04
20.00	293.15	32.92	25.62	−18.71	−17.32	−20.66	−20.59	−14.14	−13.00
25.00	298.15	31.71	24.64	−17.50	−16.11	−19.68	−19.61	−13.16	−12.02
30.00	303.15	30.56	23.70	−16.35	−14.96	−18.74	−18.67	−12.22	−11.08
35.00	308.15	29.46	22.80	−15.25	−13.86	−17.84	−17.77	−11.32	−10.18
40.00	313.15	28.42	21.95	−14.21	−12.82	−16.99	−16.92	−10.47	−9.33
45.00	318.15	27.42	21.14	−13.21	−11.82	−16.18	−16.11	−9.66	−8.52
50.00	323.15	26.47	20.37	−12.26	−10.87	−15.41	−15.34	−8.89	−7.75
55.00	328.15	25.57	19.63	−11.36	−9.97	−14.67	−14.60	−8.15	−7.01
60.00	333.15	24.70	18.93	−10.49	−9.10	−13.97	−13.90	−7.45	−6.31

**Table 5:** Depth, after applying a paleosol compaction equation [15], bulk density, and strain (ε) and elemental mass transport (τ) for mass balance calculations [8].

dZ (m)]	Depth (m)	Sample	Bulk density	Strain	SiO_2_	Al_2_O_3_	Fe_2_O_3_	Na_2_O	MgO	P_2_O_5_	K_2_O	CaO	MnO
0.20	12.10	NKT 34	2.00	0.00	0.00	0.00	0.00	0.00	0.00	0.00	0.00	0.00	0.00
0.35	12.50	NKT 36	1.96	0.01	0.00	−0.05	0.10	−0.03	−0.04	−0.01	−0.03	0.05	1.97
0.35	12.80	NKT 38	2.15	−0.05	0.05	−0.02	−0.12	0.06	−0.02	0.28	−0.06	0.09	0.02
0.40	13.20	NKT 40	1.79	0.21	0.12	−0.02	0.14	0.13	−0.03	0.08	−0.11	0.15	1.16
0.35	13.60	NKT 42	2.23	0.06	0.12	0.13	2.60	0.26	0.10	1.35	−0.21	5.18	14.89
0.25	13.90	NKT 44	1.96	0.00	−0.04	0.04	−0.03	−0.07	0.01	0.22	0.01	−0.08	0.47
0.10	14.10	NKT 46	2.04	−0.02	−0.01	−0.01	0.19	−0.09	0.10	2.00	0.04	1.00	5.50

## 2 Experimental design, materials, and methods

Seventy-five sections of the Prince Creek Formation were measured and described for grain size and sedimentary structures [2]. The NKT site (*N 69° 45.068′; W 151° 30.873′*) was selected for paleosol study based on accessibility of outcrop and abundance of paleopedological features. Macroscopic features including color, grain size, ped structure, mottles, nodules, root traces, flora and fauna were described in detail in Ref. [2]. Bulk samples were collected at 15–30 cm intervals and all samples were air-dried. Total organic carbon (TOC) was determined by Weatherford Laboratories, Shenandoah, Texas. Samples were pulverized, sieved, and reacted with concentrated HCl to dissolve carbonates. Samples were dried and combusted in a LECO model C230 combustion furnace, and CO_2_ generated by the combustion of organic matter was quantified using an infrared detector to determine TOC. Detailed description of the sampling method is given in Ref. [2], and a detailed description of geochemical processing and analytical methods is given in Ref. [1].

### 2.1 *δ*^13^C analyses of pollen grains

Sediment samples were mechanically disaggregated and treated with 10% HCl to remove carbonate, and with 49% HF to remove silicates. Samples were washed with de-ionized water several times to neutralize the acid. The final wash was through a 250 μm sieve. Sodium polytungstate was used as a heavy liquid to separate the organic fraction from remaining minerals. After freeze-drying, pollen samples were weighed for *δ*^13^C analyses. The C and N analyses (Table 1) were conducted at the Alaska Stable Isotope Facility (ASIF), University of Alaska Fairbanks. *δ*^13^C was measured using EA-IRMS. This method utilizes a Costech Elemental Analyzer (ESC 4010), and Thermo Conflo III interface with a DeltaV Mass Spectrometer [1].

### 2.2 XRF analyses

Samples were prepared by powdering using hardened steel vials from SPEX CertiPrep Group, and pressed into 35 mm diameter pellets using a polyvinyl alcohol binder. Abundances (in wt. %) of the light major oxides (SiO_2_, Al_2_O_3_, Fe_3_O_3_, Na_2_O, MgO, P_2_O_5_, K_2_O, CaO, MnO and TiO_2_) (Table 2) were measured from bulk samples using a PANalytical Axios wavelength-dispersive X-ray fluorescence spectrometer (WD-XRF) at the University of Alaska Fairbanks Advanced Instrumentation Laboratory (AIL) [1]. The chemical index of alteration minus potassium (CIA-K) was calculated according to Ref. [11] (Table 2).

### 2.3 *δ*^18^O analyses of clay samples

The total clay (<2 μm fraction) was separated using the hydrometer method. After mixing the slurry in a settling column, we used a settling time of 23 h 16 min for a 30 cm settling column at T = 20 °C room temperature. We used a pipette to siphon the supernatant into the centrifuge tubes. Then the <0.2 μm clay fraction was separated by centrifuging for approximately 6 minutes at 11,000 rpm, where the time and speed was calculated with Centriset, a USGS program, which computes settling velocity based on Stokes Law for gravitational procedures. All samples (the <2 μm and <0.2 μm fractions) were freeze-dried for stable isotope analysis. *δ*^18^O values (Table 3) were measured using a Micromass Optima dual-inlet, IRMS in the Laboratory for Stable Isotope Science at the University of Western Ontario, London, Canada [1].

### 2.4 Meteoric water composition data

We determined meteoric water composition (Table 3; Fig. 1) using the relationship that describes the oxygen isotope fractionation between smectite and water [4]. Table 4 and Fig. 1, show the relationship between meteoric water composition and temperature for maximum and minimum smectite *δ*^18^O values and compare these data with previous studies of meteoric water composition calculated from siderite [5] using the equation of [12].

### 2.5 Mean annual precipitation (MAP) data

We calculated MAP values (Fig. 2) following equations [6,7], and [13] (details in Ref. [1]).

### 2.6 Mass balance and total flux of CO_2_ data

Mass balance calculations [8] are shown in Table 5 and Fig. 3. Fig. 4 indicates the total CO_2_ flux calculated from mass balance [9,10] that was used to determine silicate weathering (the moles of CO_2_ that react as carbonic acid to release K, Ca, Mg, and Na base cations).

## References

[1] Salazar-Jaramillo S., McCarthy P.J., Ochoa A., Fowell S.J., Longstaffe F.J. (2019). Paleoclimate reconstruction of the Prince Creek Formation, Arctic Alaska, during Maastrichtian global warming. Palaeogeogr. Palaeoclimatol. Palaeoecol..

[2] Flaig P.P., Mccarthy P.J., Fiorillo A.R., Driese S.G., Nordt L.C. (2013). Anatomy, evolution and Paleoenvironmental interpretation of an Ancient Arctic Coastal plain: integrated Paleopedology and Palynology from the upper Cretaceous (Maastrichtian) Prince Creek formation, North Slope, Alaska.

[3] Fedo C.M., Nesbitt H.W., Young G.M. (1995). Unravelling the effects of potassium metasomatism in sedimentary rocks and paleosols, with implications for paleoweathering conditions and provenance. Geology.

[4] Sheppard S.M.F., Gilg H.A. (1996). Stable Isotope geochemistry of clay minerals. Clay Miner..

[5] Suarez M.B., Ludvigson G.A., González L.A., Al-Suwaidi A.H., You H.-L., Bojar A.-V., Melinte-Dobrinescu M.C., Smit J. (2013). Stable isotope chemostratigraphy in lacustrine strata of the Xiagou formation, Gansu province, NW China.

[6] Kohn M.J. (2010). Carbon isotope compositions of terrestrial C3 plants as indicators of (paleo)ecology and (paleo)climate. Proc. Natl. Acad. Sci. U. S. A..

[7] Diefendorf A.F., Mueller K.E., Wing S.L., Koch P.L., Freeman K.H. (2010). Global patterns in leaf 13C discrimination and implications for studies of past and future climate. Proc. Natl. Acad. Sci. U. S. A..

[8] Brimhall G.H., Christopher J.L., Ford C., Bratt J., Taylor G., Warin O. (1991). Quantitative geochemical approach to pedogenesis: importance of parent material reduction, volumetric expansion, and eolian influx in lateritization. Geoderma.

[9] Holland H.D., Zbinden E.A., Meybeck M. (1988). Paleosols and the Evolution of the Atmosphere Part I. Lerman A.

[10] Sheldon N.D., Tabor N.J. (2009). Quantitative paleoenvironmental and paleoclimatic reconstruction using paleosols. Earth Sci. Rev..

[11] Maynard J.B. (1992). Chemistry of modern soils as a guide to interpreting precambrian paleosols. J. Geol..

[12] Carothers W.W., Adami L.H., Rosenbauer R.J. (1988). Experimental oxygen isotope fractionation between siderite-water and phosphoric acid liberated CO2-siderite. Geochim. Cosmochim. Acta.

[13] Sheldon N.D., Retallack G.J., Tanaka S. (2002). Geochemical Climofunctions from North American soils and application to paleosols across the Eocene-Oligocene Boundary in Oregon. J. Geol..

[14] Spicer R.A., Herman A.B. (2010). The late Cretaceous environment of the arctic: a quantitative reassessment based on plant fossils. Palaeogeogr. Palaeoclimatol. Palaeoecol..

[15] Sheldon N.D., Retallack G.J. (2001). Equation for compaction of paleosols due to burial. Geology.

